# Correlation between HDL2, HDL3 and serum ferritin levels with fatty liver and NAFLD activity score (NAS) in liver histology of organ donors

**DOI:** 10.1186/s12876-021-01958-4

**Published:** 2021-10-27

**Authors:** Saman Nikeghbalian, Rasoul Rahimi, Hamed Nikoupour, Neda Soleimani, Sina Vakili, Fatemeh Zal, Fahimeh Kaveh Baghbahadorani

**Affiliations:** 1 Department of Hepatobiliary and Transplantation Surgery, Shiraz Organ Transplant Center, Shiraz University of Medical Sciences, Shiraz, Iran; 2 Namazi Hospital, Shiraz University of Medical Sciences, Shiraz, Iran; 3 Shiraz University of Medical Sciences, Shiraz, Iran; 4 Department of Pathology, School of Medicine, Shiraz University of Medical Sciences, Shiraz, Iran; 5 Biochemistry Department, Medical School, Shiraz University of Medical Sciences, Shiraz, Iran; 6 Department of Biochemistry, Shiraz University of Medical Sciences, Shiraz, Iran; 7 Maternal-Fetal Medicine Research Center, Shiraz University of Medical Sciences, Shiraz, Iran

**Keywords:** High-density lipoprotein, Nonalcoholic fatty liver disease, Ferritin, Liver transplant

## Abstract

**Background:**

Nonalcoholic fatty liver disease (NAFLD) is one of the most important liver diseases. High-density lipoprotein (HDL) has anti-atherogenic properties and its reduction can be associated with fatty liver. Serum ferritin levels are usually elevated in patients with NAFLD. This study aimed to evaluate the correlation between HDL subtypes and serum ferritin levels with evidence of NAFLD in liver histology of organ donors.

**Methods:**

One hundred organ donor patients who were eligible for the study were included in the study and ferritin; HDL2 and HDL3 were measured in blood samples. Donated liver tissue biopsy specimens were evaluated for fatty liver and NAFLD activity score (NAS). In addition, AST and ALT were measured in recipients 24 h after transplant. All data abstracted and analyzed statistically.

**Results:**

Serum HDL2 levels and HDL2/HDL3 ratio in patients with NAS > 1 were significantly lower (*P* < 0.05). Serum levels of HDL3 and ferritin were not significantly associated with NAS >1 (*P* > 0.05). In addition, serum ferritin > 1000 ng/ml in organ donors associated with increased AST and ALT levels 24 h after transplantation in the liver organ recipient.

**Conclusions:**

Lower HDL2 values and HDL2/HDL3 ratio were associated with increased NAFLD activity score, but HDL3 and ferritin did not show such a relationship. In addition, higher levels of ferritin in organ donors may be associated with increased AST and ALT 24 h after liver transplantation in the organ recipient.

## Introduction

Non-alcoholic fatty liver disease (NAFLD) is a form of hepatic steatosis in the absence of other causes of fat accumulation in the liver (such as heavy alcohol consumption) which is one of the most important liver diseases. Patients with NAFLD have hepatic steatosis with or without inflammation and fibrosis [1].

Patients with NAFLD may have mild to moderate increases in liver enzymes (AST and ALT), although normal aminotransferases do not rule out NAFLD. The prevalence of elevated aminotransferases among patients with NAFLD is unclear. The severity of elevated aminotransferases does not predict liver inflammation or fibrosis, and normal aminotransferases levels are not a sign of normal liver histology [2–4].

Fatty liver is associated with atherosclerosis and related diseases. Low HDL levels are associated with an increased risk of atherosclerosis [2, 4]. HDL levels are not only quantitatively important but also qualitatively important, and the combination of different HDL subtypes is associated with anti-atherogenic properties. Specifically, the amount of circulating HDL2 has the most protective effect against atherosclerosis [5]. In the clinic, two types of HDL can be measured in serum (HDL2 and HDL3). HDL2 particles are larger and richer in triglycerides, and HDL3 particles are smaller. Specifically, HDL2 is atheroprotective and has a higher percentage of unsaturated fatty acids. Some studies have shown that HDL2 is more decreased in patients with fatty liver, and HDL2 is a better predictor of cardiovascular disease than total HDL [6, 7].

In recent years, the association between elevated serum ferritin with metabolic syndrome and NAFLD has been suggested in many cases. Elevated ferritin levels have been observed in about 30% of patients diagnosed with NAFLD, which may be associated with elevated hepatic iron. Recent studies based on imaging with specific MRI protocols to detect iron levels in tissues have confirmed a close association between hepatocyte iron stores, steatosis, and metabolic disease [5, 8].

Nonalcoholic fatty liver disease (NAFLD) is an umbrella term for a range of liver conditions, some individuals with NAFLD can develop nonalcoholic steatohepatitis (NASH). NAFLD and NASH are actually two ends of the same spectrum. Steatosis is the accumulation of lipid droplets within hepatocytes and is considered pathologic when it affects more than 5% of hepatocytes. The difference between NAFLD and NASH is based on histological findings [9, 10].

NAFLD activity score (NAS) is a system based on histological evaluation that covers all aspects of non-alcoholic fatty liver disease and can be effective in designing treatment in both children and adults. [11].

## Materials and methods

We conducted the study between October 2019 and November 2020. This study was approved by the ethics committee of Shiraz University of Medical Sciences (ethics code: IR.SUMS.MED.REC.1399.328) and written informed consent was obtained from family of all participants in the study.

In this study, the relationship between serum levels of HDL2, HDL3 and ferritin with liver biopsy results (based on NAS) in organ donor patients was investigated. The study design was based on the possible role of lipid profile disorders and ferritin in NAFLD pathology. One of the main reasons for designing the study was the possibility of examining the liver biopsy in the donated organ.

Patients who were diagnosed as brain death and candidates for organ donation entered the study after fulfilling all the conditions for organ donation and if they met the inclusion criteria. The inclusion criteria in this study were: (1) Negative test results for HBV (hepatitis B virus), HCV (hepatitis C virus) and HIV (human immunodeficiency virus), (2) No previous history of liver cirrhosis or liver problems, (3) No previous history of hyperlipidemia. Exclusion criteria in this study was Evidence of mass or other liver disease on liver biopsy.

After reviewing the inclusion criteria, 100 patients who were diagnosed as brain death and candidate for organ donation were included in the study. Information about each patient, including age, sex, medical history, history of previous and current medications and the cause of brain death were recorded in a checklist for each patient. In this study, according to the specific conditions of organ donor brain death patients, BMI estimation was performed using mid upper arm circumference (MUAC). Table 1 shows how to estimate BMI using MUAC.

**Table 1:** Estimate BMI using mid upper arm circumference (MUAC) [21]

Mid upper arm circumference (MUAC) (cm)	Estimated body mass index (BMI) (kg/m^2^)
< 23.5	< 20
> 32.0	> 30

Before performing Harvest operation, 10 cc of blood was taken to examine HDL subtypes and ferritin level. To measure the amounts of HDL2 and HDL3 in circulation, 1.5 cc of serum was ultracentrifuged and the pattern of HDL subgroups was examined in a single step by adding heparin/Mn/DS reagent to simultaneously precipitate both the apoB-containing lipoproteins and HDL2. The reagent consisted of heparin (1071 U/ml), MnCl2 (98.7 mg/ml), and DS (12 mg/ml). The precipitation reagent (0.06 ml) was added to 0.3 ml of serum, mixed, left at room temperature for 30 min, and centrifuged at 10,000 rpm for 10 min at 4° centigrade. An aliquot of the supernatant was taken for HDL3-C measurement. HDL-C in the supernatant was determined by HDL-C assay kit (parsazmoon, iran). The measured value for total HDL3-C was multiplied by 1.2 to correct for dilution by the reagents. The value for HDL2-C was calculated as the difference between the total HDL-C (directly determined in the serum by Parsazmoon kit) and HDL3-C (All HDL assays were performed using Mindray BS200 auto-analyzer (China)). Serum ferritin was measured by Chemiluminescence immunoassay (CLIA) method. In addition, the same pathologist examined tissue biopsy of all donated livers and the fatty liver criteria and NAFLD Activity Score (NAS) were examined.

NAS scores range from 0 to 8 and do not include fibrosis. In fact, NAS include individual biopsy scores for steatosis (0–3), lobular inflammation (0–3), and hepatocellular ballooning (0–2). A score of 0–2 is seen in people who are not diagnosed with NASH, a score of 3 to 4 includes people without NASH, people with borderline diagnosis and people diagnosed as NASH, and a score of 5 to 8 includes those that considered as NASH [11].

Scoring in NAS is shown in Table 2.

**Table 2:** Scoring in NAFLD activity score (NAS) [22, 23]

Histological manifestations	Score	Criteria
Steatosis	0	< 5%
	1	5–33%
	2	33–66%
	3	> 66%
Lobular inflammation	0	None
	1	< 2 foci per 200X field
	2	2–4 foci per 200X field
	3	> 4 foci per 200X field
Ballooning	0	None
	1	A few
	2	Present in many cells

To investigate the relationship between serum levels of HDL2, HDL3 and ferritin with NAS, all participants in the study were divided into two groups:

Group 1: cases with NAS ≤ 1.

Group 2: cases with NAS > 1.

In addition, in cases where the donated liver was suitable for transplantation and liver transplantation was performed, AST and ALT were measured in the liver recipient 24 h after receiving the organ and recorded in the relevant form.

Experimental results, pathology results of liver biopsy specimens and basic information were recorded in a checklist for each patient and finally analyzed by SPSS software. Mean ± SD and frequency (relative frequency) were used to describe the quantitative and qualitative variables, respectively. Kolmogorov–Smirnov normality test and independent t test were used to analyze the data (non-parametric test was used when necessary). Statistical analysis was performed using SPSS. v. 22. Statistical significance was defined as *P* < 0.05.

## Results

At the beginning of the study, One hundred organ donors were included in the study. Out of 100 donated liver organs, 89 were suitable for liver transplantation and transplanted to the appropriate recipients. Eleven donated livers were unsuitable for transplantation, six due to severe fatty liver and 5 due to necrosis. Of the 89 organ recipients, one died less than 24 h after receiving the transplant. In the remaining 88 cases, AST and ALT were measured in recipients 24 h after transplantation.

Table 3 shows how BMI is distributed between the two groups.

**Table 3:** Body mass index (BMI) distribution between the two groups (group 1: NAS ≤ 1, group 2: NAS > 1)

BMI^a^ (kg/m^2^)	Group1: NAS^b^ ≤ 1 N = 77	Group2: NAS > 1 N = 23
BMI < 20	24 (31%)	8 (34%)
BMI = 20–30	51 (66%)	9 (39%)
BMI > 30	2 (2.6%)	6 (26%)
N	77 (100%)	23 (100%)

^a^Body mass index

^b^Non-alcoholic fatty liver disease (NAFLD) activity score

Table 4 shows the demographic characteristics and values of HDL2, HDL3, and ferritin measured and statistical analysis in two groups.

**Table 4:** Demographic characteristics and measured values ​​in the two groups (group 1: NAS ≤ 1, group 2: NAS > 1)

Parameter	Group 1: NAS^a^ ≤ 1 N = 77 Mean ± SD	Group 2: NAS > 1 N = 23 Mean ± SD	*P* value
Age	31.84 ± 10.51	32.95 ± 211.73	0.612
HDL2	12.75 ± 2.53	7.62 ± 2.36	0.001*
HDL3	22.61 ± 4.77	23.82 ± 5.43	0.329
HDL2/HDL3	0.57 ± 0.15	0.22 ± 0.07	0.001*
Ferritin	1040.20 ± 350.50	1432.30 ± 438.40	0.103

*P < 0.05 was considered statistically significant

^a^Non-alcoholic fatty liver disease (NAFLD) activity score

As shown in Table 4, HDL2 values and HDL2/HDL3 ratio in group1 (NAS ≤ 1) were significantly higher (*P* < 0.05). There was no significant difference between the two groups in terms of age, HDL3 and ferritin.

In this study, the ferritin levels measured in organ donors were highly variable (120.3–3083.4). Therefore, in order to investigate the relationship between serum ferritin levels in organ donors and AST and ALT levels in organ recipients, ferritin levels were divided into two categories: ≤ 1000 ng/ml and > 1000 ng/ml.

Table 5 shows the relationship between ferritin levels in organ donors and AST and ALT in 88 recipients 24 h after transplantation. The results showed that ferritin levels greater than 1000 ng/ml in organ donors were associated with an increase in AST and ALT levels 24 h after transplantation in the recipients.

**Table 5:** Relationship between ferritin levels in organ donor and liver transaminase levels in transplant recipients

Parameter	Ferritin ≤ 1000 ng/ml N = 61	Ferritin > 1000 ng/ml N = 27	*P* value
AST	487.07 ± 124.16	911.45 ± 314.23	0.001*
ALT	518.41 ± 168.31	894.11 ± 290.37	0.012*

*P < 0.05 was considered statistically significant

## Discussion

NAFLD is the most common cause of chronic liver disease in Western societies and appears to be the most common indication for liver transplantation by 2030 [12].

Various studies have suggested the role of lipid profiles in the occurrence of NAFLD. In addition, some studies have suggested that HDL subtype values in NAFLD patients differ from those in the general population [6, 7].

We found a significant decrease in HDL2 and HDL2/HDL3 ratio in patients with NAS > 1, a finding similar to that other reports. Kantartzis et al. demonstrated that decreased HDL2 values and HDL2/HDL3 ratios were strongly correlated with fatty liver detection, which was much stronger than total HDL correlation. In this study, no difference in HDL3 values ​​was observed between the two groups. However, liver biopsy was not used to diagnose NAFLD in this study [7].

In various studies, the diagnosis of NAFLD in patients has been associated with an increased risk of atherosclerosis [12, 13]. Arts et al. found that HDL2 and HDL3 levels decreased in cases of rheumatoid arthritis with atherosclerosis [13], while in the results of our study and Kantartzis et al. study, no difference was observed in the level of HDL3 in NAFLD patients.

In addition, Salonen et al. study showed that total HDL and HDL2 were inversely related to myocardial infarction but HDL3 was not significantly associated with it [14].

Normally most circulating HDL is in the form of HDL3 [15]. Similarly, in our study, circulating HDL3 levels were higher than HDL2. HDL particles are composed of 4 apolipoproteins. HDL subtypes are composed of lipoproteins Apo A-I and Apo A-II together or Apo A-I alone. HDL2 is often made of Apo A-I alone. Fadaei et al. demonstrated that Apo A-I levels are significantly lower in NAFLD patients [16]. This finding is similar to the results of our study that HDL2 levels were lower in patients with increased NAS.

Serum ferritin levels are usually elevated in patients with nonalcoholic fatty liver disease (NAFLD), due to inflammation or increased iron stores [17, 18]. Kowdley et al. found that ferritin > 450 ng/ml was associated with higher levels of NAS, AST, ALT and increased hepatic fibrosis [17]. However, in our study, there was no relationship between serum ferritin levels in organ donors and NAS in liver biopsy. To interpret these results, we must consider the role of ferritin as an acute phase reactant in organ-donor brain death patients. This could be the reason for the very different amounts of ferritin in organ donors in this study.

Hagström et al. Showed that increased ferritin levels in NAFLD patients during long-term follow-up were associated with increased NAS and hepatic fibrosis [19].

As shown in Table 5, ferritin > 1000 ng/ml in organ donors was associated with increased AST and ALT 24 h after transplantation in recipients. This may be related to the primary inflammatory process that increases ferritin in the organ donor.

Wakiya et al. demonstrated that serum ferritin > 1000 ng/ml in organ donors was associated with increased intraoperative ferritin levels in the recipient. In addition, in this study, serum ferritin > 1000 ng/ml in transplant recipients was associated with longer warm ischemia time and increased levels of hepatic transaminases after surgery. Finally, in this study, increased ferritin levels in organ donors were introduced as a predictor of ischemia/reperfusion injury in donated liver [20]. These findings are similar to the results of our study that elevated Ferritin levels in donors were associated with increased hepatic transaminases in recipients.

This study has been associated with limitations. One of the limitations was the impossibility of examining various factors in the incidence of fatty liver (due to the special conditions of patients diagnosed with brain death). On the other hand, brain dead patients are prone to increased ferritin as an inflammatory response that can affect their ferritin levels and act as a data-influencing factor.

Finally and based on our results, we can introduce HDL2 and HDL2/HDL3 as two contributing factors to predict the quality of liver pathology, especially in the case of NAFLD in organ donors. In addition, due to the limitations of liver biopsy for the diagnosis of NAFLD in the general population, it may be possible to use HDL2 and HDL3 measurements to diagnose NAFLD.

Although no correlation was found between serum ferritin levels and the pathology of liver biopsy in our study, the association between elevated ferritin in donors and elevated liver transaminases in recipients could confirm the role of ferritin as a predictor of liver damage.

## Conclusion

Taken together, these data recommend that HDL subtypes can play an important role in predicting fatty liver. Measuring HDL2 and HDL3 is an easy and accessible way to predict the quality of liver pathology in donors. This method can also be used to predict fatty liver in the general population. In addition, ferritin levels in liver donors may be used to predict liver damage and transaminase levels in transplant recipients. Although considering the disparate findings above, additional extensive studies are needed to better examine the relationship between HDL2, HDL3, serum ferritin, liver transaminases, and liver pathologies.

## Data Availability

All respectable readers and researchers can request the data by directly contacting the primary author at rahimi.rasoul78@gmail.com.

## Abbreviations

NAFLD: Nonalcoholic fatty liver disease
HDL: High-density lipoprotein
AST: Aspartate aminotransferase
ALT: Alanine transaminase
NASH: Nonalcoholic steatohepatitis
NAS: Nonalcoholic fatty liver disease activity score
VLDL: Very low density lipoproteins
LDL: Low-density lipoproteins
BMI: Body mass index
MUAC: Mid upper arm circumference
Apo: Apolipoprotein

## Declarations

Declaration of Helsinki: All methods were performed in accordance with the relevant guidelines and regulations.
